# Cryptic Subtelomeric Rearrangements and X Chromosome Mosaicism: A Study of 565 Apparently Normal Individuals with Fluorescent *In Situ* Hybridization

**DOI:** 10.1371/journal.pone.0005855

**Published:** 2009-06-10

**Authors:** Jasen L. Wise, Richard J. Crout, Daniel W. McNeil, Robert J. Weyant, Mary L. Marazita, Sharon L. Wenger

**Affiliations:** 1 Department of Pathology, West Virginia University, Morgantown, West Virginia, United States of America; 2 School of Dentistry, West Virginia University, Morgantown, West Virginia, United States of America; 3 Department of Dental Public Health and Information Management, University of Pittsburgh School of Dental Medicine, Pittsburgh, Pennsylvania, United States of America; 4 Center for Craniofacial and Dental Genetics, Departments of Oral Biology and Human Genetics, University of Pittsburgh, Pittsburgh, Pennsylvania, United States of America; Health Canada, Canada

## Abstract

Five percent of patients with unexplained mental retardation have been attributed to cryptic unbalanced subtelomeric rearrangements. Half of these affected individuals have inherited the rearrangement from a parent who is a carrier for a balanced translocation. However, the frequency of carriers for cryptic balanced translocations is unknown. To determine this frequency, 565 phenotypically normal unrelated individuals were examined for balanced subtelomeric rearrangements using Fluorescent *In Situ* hybridization (FISH) probes for all subtelomere regions. While no balanced subtelomeric rearrangements were identified, three females in this study were determined to be mosaic for the X chromosome. Mosaicism for XXX cell lines were observed in the lymphocyte cultures of 3 in 379 women (0.8%), which is a higher frequency than the 1 in 1000 (0.1%) reported for sex chromosome aneuploidies. Our findings suggest that numerical abnormalities of the X chromosome are more common in females than previously reported. Based on a review of the literature, the incidence of cryptic translocation carriers is estimated to be approximately 1/8,000, more than ten-fold higher than the frequency of visible reciprocal translocations.

## Introduction

Subtelomeres are the most distal sequences of non-repetitive DNA on the chromosome, and have the highest density of genes in the genome [1]. Any rearrangement or deletion in these gene-rich regions could have severe phenotypic consequences. Numerous studies have shown that 0.5 to 10.7% (variation due to study selection criteria) of patients with unexplained mental retardation (MR) have an unbalanced cryptic subtelomeric rearrangement or deletion [2]–[11].

Cytogenetically visible balanced translocations have an incidence of 1 in 600 in the general population [12]. Parents, who are carriers of balanced translocations, are at risk for having children with unbalanced gene complements. Fifty percent of the gametes produced by a balanced translocation carrier will have segmental aneuploidy, which can result in a child with an unbalanced rearrangement. The most likely way to identify a balanced carrier is through a child who has been identified with an unbalanced rearrangement.

Several studies have reported that half of all patients with an unbalanced cryptic rearrangement have inherited it from a parent with a cryptic balanced translocation [4], [10]. The frequency of individuals who carry a balanced cryptic translocation is unknown. In this study, 565 unrelated, phenotypically normal individuals were screened with subtelomere FISH probes to determine if balanced cryptic translocation carriers could be identified.

## Methods

Subjects for this study were drawn from the Center for Oral Health Research in Appalachia (COHRA) [13], an ongoing cross-sectional oral health etiology study. COHRA ascertains families from two central West Virginia counties and two western Pennsylvania counties and performs a detailed assessment protocol after an informed consent process approved by the Institutional Review Boards (IRB) of the University of Pittsburgh and West Virginia University (WVU). A total of 484 COHRA subjects were included in the current study: 164 male and 320 female. In addition, IRB approval was obtained to use discarded samples from the WVU cytogenetics laboratory. These 81 (22 males and 59 females) samples were selected on the basis of a normal karyotype at least the 550+ band level, which were then deidentified prior to analysis.

Peripheral blood lymphocytes were processed using standard clinical cytogenetic techniques. Cells were dropped onto slides, which were then immersed through the following series of washes in coplin jars: 2× SSC for 10 minutes at 37°C, 1% formaldehyde for 15 minutes at room temperature (RT), 1× PBS for 5 minutes at RT, pepsin solution for 13 minutes at 37°C, 1× PBS for 5 minutes at RT and then air dried. The slides were then placed through a series of ethanol washes of 70%, 85%, and 100% for 1 minute each and allowed to air dry at room temperature.

Working probe solutions were prepared by adding 3 µl of ToTelVysion probe solution (Abbott Molecular Inc, cat# 33-270000) to 30 µl of cDenHyb (InSitus, cat #D002) in a microfuge tube and mixed well. Three µl of each working probe solution was placed in the middle of one of 5 respective circled areas on a slide. A 12 mm circular coverslip was added and all air bubbles driven out. When 5 spots per slide were completed, lab tape was placed across the entire slide and pressed firmly for a tight seal. Slides were placed on a hotplate for 3 minutes at 90°C, then in a light-tight box, and incubated overnight in a 37°C water bath.

The next day, in a minimal light room, the slides were removed from the water bath and de-coverslipped. The slides were then washed in 0.4× SSC/0.3% NP-40 at 73°C for two minutes followed by 30 seconds in 2× SSC/0.1% NP-40 at room temperature. The slides were then completely air dried in the dark. Twenty µl of 1× DAPI counterstain was applied to the slides and coverslipped.

A Leica epi-fluorescent microscope equipped with a DAPI single bandpass, aqua single bandpass, and a red/green dual bandpass filter was used for signal enumeration. Yellow signals were read using the red/green filter. Five metaphase and 5 interphase cells were scored for each of 15 subtelomeric probe sets per subject.

To confirm abnormal subtelomere FISH results regarding X chromosomes, slides were prepared as described previously and hybridized with X/Y centromere probes. Two scorers analyzed 100 cells each for percentage of abnormal cells.

## Results

**Figure 1:**
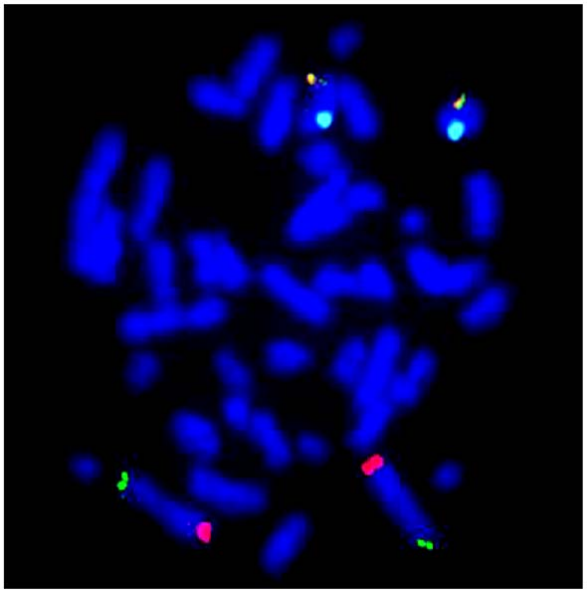
An example of a FISH hybridized metaphase spread. Four different probe signals are visible: 2p (green signal), 2q (red signal), Xq/Yq (yellow signal) and X (aqua signal). This pattern represents the pattern seen in a normal diploid cell from a female.

A total of 565 samples were evaluated for cryptic rearrangements using subtelomeric FISH probes. No balanced cryptic rearrangements were observed by FISH, all samples showing normal number and location of signals (Fig. 1).

**Figure 2:**
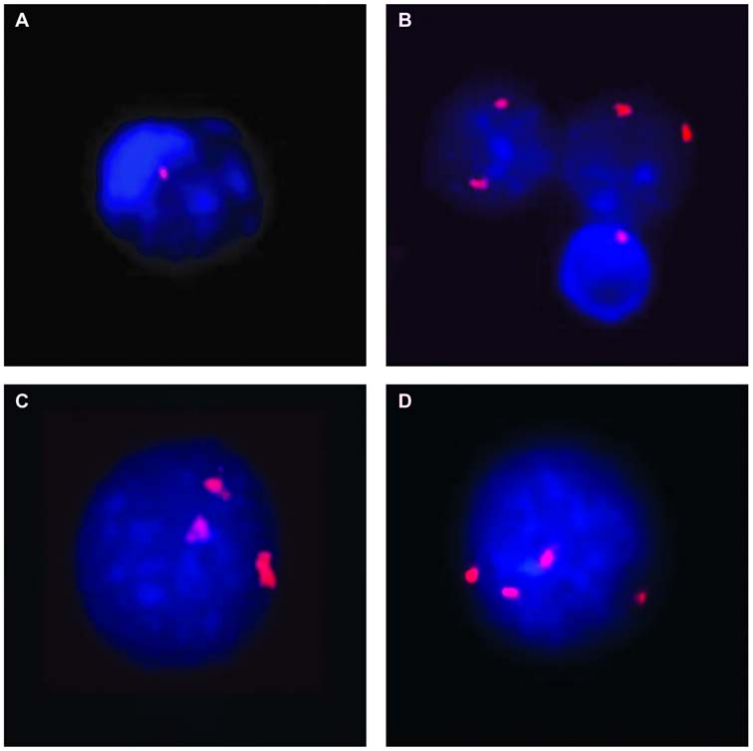
Interphase cells hybridized with a FISH probe for the centromere of the X chromosome. A) A cell with one signal for the centromere of the X chromosome or monosomy X. B) Two normal cells showing two signals for the X chromosome, and one cell with monosomy X. C) A cell showing trisomy X. D) A cell with tetrasomy X.

Among the specimens that were analyzed with subtelomeric FISH probe sets containing Xp/Yp and Xq/Yq, mosaicism for X chromosome aneuploidy was identified in 3 of 379 women (0.8%). The results were confirmed using a separate X/Y centromeric probe set. The FISH results in interphase cells for these individuals identified 89% triple X in a 36 year old, 5% triple X chromosome in a 52 year old, and multiple cell lines including 11% single X, 6% XXX and 2% XXXX in a 54 year old (Fig. 2).

## Discussion

### Subtelomeric Rearrangements

No balanced cryptic translocations were found among the 565 subjects who were screened by subtelomere FISH. From the small sample size, it was not possible to determine the frequency of balanced subtelomeric translocation carriers. Our sample size was limited due to the number of individuals enrolled in the study who donated blood, loss due to culture failures, and the cost of FISH probes.

Since the frequency for a balanced cryptic rearrangement could not be estimated from our study sample, we estimated the frequency based on reports in the literature. Carrier frequency (X) was estimated based on the equation: (A)(B)(C)(D)(E) = X where A is the 2% of the population with MR[14], B is the 50% of MR patients with unknown etiology [15], C is the estimated 5% incidence of subtelomeric abnormalities in MR patients with unknown etiology [16], D is the 50% percent risk for inheriting the unbalanced rearrangement from a parent with a balanced subtelomeric translocation [4], and E is the 50% chance that a carrier would have a child with an unbalanced rearrangement (due to chromosome segregation in the gametes).

Using this equation, the incidence of cryptic *balanced* subtelomeric rearrangement carriers in the general population is approximately 1 in 8000. Therefore, it is not surprising that the current series of 565 subjects found no one with a cryptic balanced translocation.

The reported incidence of individuals with *unbalanced* subtelomeric rearrangements in the general population has been estimated by Knight and colleagues [4] to be 2.1 in 10,000 (∼1 in 4762). Because half of these individuals inherited the rearrangement from a parent [4], [10], the frequency of parents who are *balanced* translocation carriers would be half as frequent, or around 1 in 9524. The difference between this calculation and ours is most likely due to the variation in the reported percentages and criteria for evaluation of MR. Regardless, the incidence of balanced subtelomeric rearrangements is at least 13 times more prevalent than carriers of visible reciprocal translocations.

### X Chromosome Mosaicism

Individuals who are mosaic have two or more populations of somatic cells that are genetically different. Aneuploidy of the X chromosome can arise by mitotic nondisjunction or anaphase lag. If this event occurs during early fetal development, higher percentages of mosaicism will occur. If the error occurs at a later time during fetal development or after birth, then lower percentages will be present. Individuals who have low level mosaicism for the X chromosome are less likely to have a clinically relevant phenotype and therefore would go undiagnosed in the general population. There are several factors to consider with low level mosaicism: are the results due to genuine mosaicism, technical artifact [17], or age related?

X chromosome aneuploidy has been attributed to premature centromere division in older women. Several studies have demonstrated that peripheral blood metaphase cells from women generally 50 years of age and older could have an average of 4–5% X chromosome loss and less than 1% gain attributed to mitotic error [18]–[21] This finding has been substantiated in interphase cells using FISH probes, demonstrating that women older than 60 years of age had an average X chromosome loss of 3.4% (as high as 9%) and gain of less than 1% [22]–[23].

FISH studies have shown that cultured lymphocytes reflect in vivo aneuploidy rates and that there is no significant difference between cultured lymphocyte and uncultured lymphocyte stability [22], [24]. All 3 of the individuals identified in this study to have X chromosome mosaicism had gains of X chromosomes. Although two of the three women were over the ages of 50, all three women had a cell line with 5% or greater for an extra X chromosome, suggesting that the aneuploidy is not related to age or tissue culture artifact.

We were unable to find any literature that has estimated the incidence of sex chromosome mosaicism in the general population or the incidence of balanced cryptic subtelomeric rearrangements in the general population. We estimate that the incidence of balanced cryptic translocation to be at least 1 in 8,000. To our knowledge, the finding of mosaicism in 0.8% of women may be the first reported incidence of low level sex chromosome mosaicism in the general population, much higher than the 0.1% reported in newborn studies for sex chromosome aneuploidy, which was based on analysis of 3–5 cells [25]. Our findings suggest that numerical abnormalities of the X chromosome may be more common in females than previously reported.
